# The effects of mangosteen peel (*Garcinia mangostana* L.) and Turmeric (*Curcuma domestica* Val) flour dietary supplementation on the growth performance, lipid profile, and abdominal fat content in Cihateup ducks

**DOI:** 10.14202/vetworld.2019.402-408

**Published:** 2019-03-15

**Authors:** Andri Kusmayadi, Kamiel Roesman Bachtiar, Caribu Hadi Prayitno

**Affiliations:** 1 Department of Animal Science, Faculty of Agriculture, Universitas Perjuangan, Tasikmalaya 46115, West Java, Indonesia; 2 Department of Pharmacy, Faculty of Health Science, Universitas Perjuangan, Tasikmalaya 46115, West Java, Indonesia; 3 Department of Feed Science, Faculty of Animal Science, Universitas Jenderal Soedirman, Purwokerto 53123, Central Java, Indonesia

**Keywords:** Cihateup duck, lipid profile, mangosteen peel flour, performance, turmeric flour

## Abstract

**Background:**

Bioactive compounds in mangosteen peel and turmeric have been shown to possess antioxidant and hypolipidemic properties.

**Aim:**

This study aimed to examine the effect of mangosteen peel flour (MF) and turmeric flour (TF) dietary supplementation on the performance, lipid profile, and abdominal fat content of Cihateup ducks.

**Materials and Methods:**

The study was conducted for 56 days (8 weeks) using 84-day-old male Cihateup ducks that were allocated into seven treatments with three replications and each containing four ducks as subreplicates. The seven groups were positive control, containing 100% of basal ration/BR + 50 ppm bacitracin (R_0_), BR + 2% MF (R_1_), BR + 1.5% MF + 0.5% TF (R_2_), BR + 1% MF + 1% TF (R_3_), BR + 0.5% MF + 1.5% TF (R_4_), BR + 2% TF (R_5_), and BR only as negative control (R_6_). The data of each variable were analyzed using a completely randomized design (one way). Significant differences (p<0.05) were determined with Duncan test.

**Results:**

The results indicated that the addition of MF and TF significantly affected (p<0.05) body weight, weight gain, mortality rate, low-density lipoprotein, high-density lipoprotein, and abdominal fat levels. At the same time, MF and TF significantly influenced (p<0.01) total cholesterol and triglyceride concentration. Non-significant differences (p>0.05) in feed intake and feed conversion of Cihateup ducks were observed.

**Conclusion:**

The combination of MF and TF at a balanced ratio (R_3_) can be used as an alternative feed additive to improve performance, lipid profile, and abdominal fat of Cihateup ducks.

## Introduction

Cihateup duck is local poultry species from Tasikmalaya, West Java, Indonesia, which serves both as meat and egg producer. Cihateup duck has slower growth rates, worse feed conversion rate, and carcass with higher fat content [1] and high blood cholesterol levels [2] compared to broilers. Therefore, the current research is focused on feed additives that can improve growth performance with a concomitant reduction of fat content in duck carcass. In order to overcome this problem, farmers usually use synthetic antibiotics. The application of synthetic antibiotics is very effective in giving a boost to growth but has a negative impact on consumer’s health such as resistance to certain antibiotics, carcinogenic responses (cancer risk), and residues in the derived products. The effects of synthetic antibiotic residues in food can cause transfer of resistant bacteria to the human organism, immunological effects, carcinogenic, mutagenic, hepatotoxic, reproductive disorders, and allergies [3].

The herbal feed additives could serve as an alternative to antibiotics because they have many benefits and are natural, safe, and environmentally friendly [4]. Turmeric is an herb that has the potential to improve livestock growth while reducing fat level in carcass. Broiler chicks fed turmeric flour (TF) had increased weight gain, improved energy efficiency, and feed conversion ratio (FCR) [5,6]. In addition, turmeric also has a good impact on improving the blood lipid profile of poultry [7]. Curcuminoid substances possess antibacterial properties and can stimulate the gallbladder wall to secrete bile so that it can facilitate fat metabolism [8,9]. At the same time, mangosteen peel contains xanthones exerting hypolipidemic [10] and hypocholesterolemic [11] activities. This decrease in blood cholesterol levels is caused by inhibition of cholesterol synthesis in the body [11]. In addition, mangosteen peel contains high levels of antioxidants [12] and can reduce the stress level of ducks. Apart from xanthone, mangosteen peel contains α-mangosteen, a pigment that can improve secretion of pancreatic lipase and α-amylase; enzymes that play a significant role in antiobesity mechanism [10].

Apart from turmeric, mangosteen peel could serve as a supplement of duck diets. The use of these agents (both turmeric and mangosteen peel) has never been tested in Cihateup ducks although both possess properties that support and complement each other. Therefore, this study aimed to examine the effect of mangosteen peel flour (MF) and TF dietary supplementation on the performance, lipid profile, and abdominal fat content of Cihateup ducks.

## Materials and Methods

### Ethical approval

The use of material (ducks) in this study has been approved by the Animal Ethics Committee for Using Animal and Scientific Procedures in Faculty of Animal Science, Universitas Padjadjaran, Indonesia.

### Animal and treatments

Selection was done by using a simple method involving several five senses to choose male ducks only from hatchery (recommended by duck breeder expert). Male ducks can be selected easily using (1) the sense of sight to observe male genitals which are characterized by a slight bulge, the color of the fur that looks more dark brown and body posture that tends to be larger than the female duck; (2) the sense of hearing to observe the sound of male ducks marked with a voice that is more hoarse and less loud. The 84 male Cihateup ducks of day old were assigned into seven treatment groups, namely positive control containing 100% basal ration/BR + 50 ppm bacitracin (R_0_), BR + 2% MF (R_1_), BR + 1.5% MF + 0.5% TF (R_2_), BR + 1% MF + 1% TF (R_3_), BR + 0.5% MF + 1.5% TF (R_4_), BR + 2% TF (R_5_), and BR only as negative control (R_6_). The dietary supplementation with MF and TF started from the 2^nd^ week until the end of the maintenance period (8 weeks). Drinking water was provided ad libitum. The diet composition and analysis are presented in Table-1.

**Table-1:** Diet composition and analysis.

Feed composition	Period (%)	

	Starter (0-3 weeks)	Grower (4-8 weeks)
Corn	29.80	26.00
Soybean meal	8.54	9.00
Bran	33.50	33.00
Fish meal	8.54	9.00
Basal ration	18.40	21.27
Essential oil	0.50	0.70
Salt	0.15	0.15
Dicalcium phosphate	0.50	0.50
Premix	0.07	0.07
Methionine	0.03	0.08
Nutrient (%)		
Water content	11.00	12.00
Protein	18.70	15.40
Crude fat	6.88	5.88
Crude fiber	5.15	5.45
Ash	6.50	6.50
Ca	0.72	0.72
P	0.42	0.36
Methionine+Cystine	0.69	0.57
Lysine	1.10	0.90
Energy (Kcal EM/kg)	2900	2900

### Performance measurements

Cihateup ducks were weighed every week to measure weekly (UN) weight gain. Feed intake was daily recorded. Feed remnants were measured for the estimation of intake. Feed conversion was calculated by dividing feed intake with body weight (BW) gain. Mortality rate was also measured by counting the number of died ducks during the experimental period.

### Lipid profile and abdominal fat level determination

Blood samples of Cihateup duck taken through the brachial vein and analyzed to determine cholesterol, triglycerides, high-density lipoprotein (HDL), and low-density lipoprotein (LDL) levels. Blood samples of Cihateup duck were collected through the brachial vein. Blood was collected in ethylenediaminetetraacetic acid tubes. Then, it was stored in a cooler box. Then, it was stored in a cooler box and was centrifuged at 4000 rpm for 10 min to obtain blood plasma. After this procedure, blood plasma was removed in the Eppendorf tube and put into the freezer before analyses. Cholesterol levels were determined by the CHOD-PAP enzymatic colorimetric method, triglyceride levels were determined with the GPO-PAP method, and HDL analysis was determined using the enzymatic colorimetric method after precipitation of β-lipoprotein with phosphotungstate and magnesium chloride. LDL levels were obtained using formula LDL = Total cholesterol - HDL - 1/5 Triglycerides. Abdominal fat levels were calculated by comparing the weight of abdominal fat and the live weight of ducks.

### Statistical analysis

The data obtained were processed using a completely randomized design (one way). Significant differences (p<0.01 or p<0.05) were determined with Duncan test.

## Results and Discussion

### BW

Based on the data presented in Table-2, dietary supplementation with MF and TF significantly increased (p<0.05) BW of Cihateup ducks compared to negative control group. The combination of MF and TF (R_3_; 1% and 1% and R_4_; 0.5% and 1.5%, respectively) showed the best results compared to controls and other treatments. The results of the present study are in accordance with that of Ürüşan and Bölükbaşı [13] who report that the addition of TF at the level of 10 g/kg (1%) in the diet has a significant effect (p<0.05) on the broiler BW. The addition of MF combined with ginger at the level of 1% significantly influences (p<0.05) the BW of broilers that are reared under heat stress [11]. Similar results are reported by Palapol *et al*. [14] who found that the use of mangosteen peel improves the performance and BWs of broilers.

**Table-2:** Average performance of Cihateup ducks given ration contains mangosteen peel flour and turmeric flour of research results.

Treatments	Body weight (g)	Weight gain (g)	Feed intake (g) (ns)	Feed conversion (ns)	Mortality (%)
R_0_(Positive control)	1453.25±68.13^b^	923.46±23.56^b^	4464.03±103.24	5.07±0.56	0.00±0.00^a^
R_1_(2% MF)	1457.56±57.67^b^	885.34±41.14^ab^	4437.17±117.65	5.21±0.87	1.39±0.20^b^
R_2_(1.5% MF+0.5% TF)	1453.56±39.22^b^	877.34±33.87^ab^	4445.75±98.26	5.11±0.45	0.00±0.00^a^
R_3_(1% MF+1% TF)	1568.87±98.76^c^	985.00±65.43^c^	4469.00±138.27	5.03±0.23	0.00±0.00^a^
R_4_(0.5% MF+1.5% TF)	1534.33±54.41^c^	948.46±57.76^b^	4427.33±156.59	5.08±0.44	0.00±0.00^a^
R_5_(2% TF)	1473.44±35.23^b^	974.66±34.28^c^	4481.67±115.30	5.12±0.38	0.00±0.00^a^
R_6_(Negative control)	1293.75±43.79^a^	776.49±36.24^a^	4334.67±100.45	5.24±1.03	2.74±0.34^b^

Description-Superscript different within the same column shows a significant difference, ns=Non significant, MF=Mangosteen peel flour, TF=Turmeric flour

This study confirms that the consumption of curcumin in turmeric and xanthone in mangosteen peel by the livestock results in an increase of the BW in Cihateup ducks. The addition of these herbal ingredients in a balanced ratio has a greater impact than the single incorporation of them or the other combinations of herbs. Turmeric and mangosteen peel play a significant role in increasing the effectiveness of the digestive system by balancing the intestinal microflora population through their antimicrobial properties [15] and as a result, an increased BW is observed.

### Weight gain

The data in Table-2 show that supplementation of MF and TF significantly affect (p<0.05) weight gain of Cihateup ducks. R_3_ and R_5_ groups had the greatest values among the experimental groups. This result is consistent with the findings of Mondal *et al*. [16] who state that the addition of TF in broiler feed has a significant effect (p<0.05) on the weight gain, feed conversion, and decreased the levels of abdominal fat (p<0.01). This happens because the antioxidant activity of mangosteen peel and turmeric is optimal at the level of 1% so that it can stimulate the synthesis of enzymes and proteins in the digestive system of poultry, especially the secretion of endogenous digestive enzymes [17]. In addition, bioactive compounds in these herbal ingredients play a role in reducing intestinal viscosity so that they are positively correlated with nutrient absorption and ultimately have a significant effect on weight gain.

In another study, the addition of TF and MF did not have a significant effect on weight gain [18]. They reported that turmeric dietary supplementation at various levels (0.25, 0.5, 0.75, and 1%) had no significant effects on the weight gain of broilers. Ratika *et al*. [19] further reported that the addition of 0.5% of turmeric in the diet did not have a significant effect on broiler growth performance. On the other hand, TF at the level of 2% affected weight gain in rabbits [20].

### Feed intake

The data in Table-2 show that the supplementation of MF and TF does not have a significant effect (p>0.05) on the feed intake of Cihateup duck. These findings are in accordance with the previous studies which state that at the same time, the addition of turmeric at the level of 1.0, 2.0, and 3.0% has no significant effect on feed intake, egg weight, and chicken egg production level [21,22]. The other studies report that the addition of MF to Mojosari ducks at the level of 1.5% does not affect feed intake, weight gain, and feed conversion [23]. Although not shown in the present study, several bioactive compounds in herbal plants are able to stimulate appetite and increase feed intake and secretion of endogenous digestive enzymes, activate immune responses, antioxidants, and antimicrobials [24].

### FCR

Table-2 shows that MF and TF had no significant effect (p>0.05) on the feed conversion. This is consistent with previous literature by Emadi and Kermanshahi [25] which reports that the addition of turmeric at the level of 2.5, 5, and 7.5 g/kg of feed does not affect the BW and feed conversion of broiler chickens. Meanwhile, 5 g of turmeric per kg of feed does not affect the BW and feed conversion of broiler chickens although has an impact on the feed consumption and conversion from the starter period to the finisher period [26]. On the other hand, there are studies that show a negative impact of turmeric at the level of 1% on feed intake, weight gain, and feed conversion [27]. Moreover, Attia [22] reported that the addition of turmeric combined with other herbs at a balanced level (0.15:0.15) has a significant influence on the feed conversion parameters. In this case, herbal bioactive compounds play a role in regulating the amount of feed intake, affecting the microflora to reduce the activity of pathogenic bacteria in absorbing feed nutrients.

### Mortality rate

The mortality values presented in Table-2 show that supplementation of MF and TF significantly affected (p<0.05) mortality rate of Cihateup ducks. This study is in accordance with the report from Samarasinghe *et al*. [21] which states that the use of herbs as feed additives in broiler diets shows a great efficiency as antimicrobial agents, especially against *Escherichia* *coli* and coliform bacteria in the duodenum. Ürüşan and Bölükbaşı [13] also report that TF at a level of 0.6-1% could significantly (p<0.01) reduce *E. coli* counts in jejunum. On the other hand, the addition of turmeric at a higher level (2%) can increase the amount of lactic acid bacteria which is very useful as a natural probiotic.

The data in Table-2 prove that all treatments containing turmeric have zero mortality because turmeric could also act as an immunomodulator [28] and improve blood biochemical parameters, livestock health status, and nutrient metabolism in the body [29]. This is also supported by the role of turmeric as an antioxidant compound; cattle that are challenged with aflatoxin B1 are more resistant to stress after turmeric supplementation by reducing the production of free radicals [30]. The increased antioxidant activity is also related to the increased glutathione peroxidase and superoxide dismutase activities as shown in broilers that are kept under stress conditions [31].

### Lipid profiles

#### Total cholesterol

The effect of supplementation of the MF and TF on total cholesterol levels is presented in Table-3. The use of mangosteen peel and TF (R_1_, R_2_, and R_3_ groups) has a significant effect (p<0.01) on total blood cholesterol levels in Cihateup ducks compared to that of R_6_ group. The combination treatment of MF and TF at the level of 1.5% + 0.5% shows the lower values compared to the other treatments. These results are consistent with a previous study which states that the addition of herbal combination of TF and thyme can reduce the level of total cholesterol, HDL, LDL, and blood triglycerides in broiler chickens [6]. Total cholesterol level can also be decreased by adding a combination of mangosteen peel with ginger [11]. Xanthones have a hypocholesterolemic activity which is able to reduce cholesterol levels [32].

**Table-3:** Average lipid profiles of Cihateup ducks given ration contain mangosteen peel flour and turmeric flour of research results.

Treatments	Lipid profiles			

	Total cholesterol*	Triglycerides*	LDL**	HDL**
R_0_(Positive control)	143.80±36.34^b^	81.80±4.95^b^	47.70±4.59^ab^	62.65±7.35^b^
R_1_(2% MF)	146.50±4.24^b^	96.30±53.88^c^	46.40±3.61^ab^	58.95±4.95^ab^
R_2_(1.5% MF±0.5% TF)	125.45±5.73^a^	93.70±17.82^c^	40.40±4.38^a^	54.50±1.98^a^
R_3_(1% MF±1% TF)	132.75±20.72^ab^	59.65±11.53^a^	44.35±9.12^ab^	56.55±6.58^ab^
R_4_(0.5% MF±1.5% TF)	151.25±27.22^bc^	81.35±17.18^b^	49.65±10.67^b^	65.55±9.55^c^
R_5_(2% TF)	152.30±22.20^bc^	87.95±37.12^bc^	51.30±11.68^b^	68.35±7.07^c^
R_6_(Negative control)	160.71±18.56^c^	92.27±24.39^c^	45.78±6.34^ab^	67.16±6.54^c^

Description -

* Superscript different within the same column shows a significant difference (p<0.01).

** Superscript different within the same column shows a significant difference (p<0.05). LDL=Low-density lipoprotein, HDL=High-density lipoprotein, MF=Mangosteen peel flour, TF=Turmeric flour

Adiputro *et al*. [33] reported that the reduction in blood cholesterol levels is observed as a result of the inhibition of cholesterol formation. During the formation of cholesterol, there is a stage of squalene synthesis. At this stage, the formation of the two molecules of farnesyl pyrophosphate and elimination of pyrophosphate radicals occur. The formation of the two molecules of farnesyl pyrophosphate is characterized by the production of two farnesyl pyrophosphate radicals. This production is inhibited by the antioxidant properties of the ethanolic extract of mangosteen pericarp, thus inhibiting squalene formation. Chomnawang *et al*. [34] also demonstrated the potential of mangosteen pericarp to inhibit superoxide radicals through the mechanism of the transfer of electrons or hydrogen atoms. Concerning the mechanism of increased cholesterol catabolism, cholesterol is converted into bile salts. The process of conversion of cholesterol into bile salts requires the availability of oxygen, NADPH, and cytochrome p450. Therefore, the mechanism of cholesterol reduction by mangosteen pericarp in the present study was performed through the inhibition of cholesterol synthesis [33].

#### Triglyceride

The data in Table-3 show that the supplementation with MF and TF plays a significant role in reducing blood triglyceride levels (R_3_ and R_4_ group) (p<0.01). The combination treatment of MF and TF at R_3_ and R_4_ shows the best results compared to other treatments. The data are in accordance with a previous study reporting that the combination of turmeric and thyme has a significant effect on decreasing blood triglyceride level due to a reduction in enzyme activity synthesis [6]. The other factors are that curcumin in turmeric possesses hypolipidemic properties that cause not only a decrease of triglycerides but also of total cholesterol, HDL, and LDL [6]. Likewise, another study by Hidanah *et al*. [11] reports that broiler’s blood triglyceride level can be decreased by adding a combination of mangosteen peel with ginger. The combination of mangosteen peel and turmeric has a better effect on decreasing blood triglyceride level than other treatments. The reduction in triglyceride levels in the present study (Table-3) was possibly caused by the increased very LDL (VLDL) and chylomicron catabolism. VLDL is a triglyceride transporter originating in the liver, while chylomicrons are triglyceride transporters from the intestines. VLDL and chylomicron catabolism are affected by lipoprotein lipases that hydrolyze triglycerides into free fatty acids and glycerol [33].

#### LDL

The supplementation of MF and TF significantly affect (p<0.05) LDL blood level of Cihateup ducks. The treatment of R_2_ containing 1.5 and 0.5% combination of mangosteen peel and TF shows the best results compared to other treatments. This result is in accordance with a previous study of Kermanshashi and Riasi [35] who report that the addition of turmeric at the level of 0.5-1.5 g/kg BW in ration can reduce LDL level, triglycerides, and total cholesterol in the blood. This is because turmeric has strong properties to change lipid profile [36]. The addition of 5 g/kg BW of turmeric can also increase hemoglobin and red blood cells and reduce LDL and VLDL while using 2.5 g/kg BW of feed can increase HDL level and broiler total cholesterol [25].

A higher proportion of lipids to proteins in the lipoproteins is associated with a lower density of lipoproteins. Thus, the higher the LDL levels, the lower the HDL levels. Furthermore, increases in triglyceride levels lead to increases in chylomicron and VLDL levels, as transporters of triglycerides. LDL is the last stage of VLDL catabolism; therefore, raised VLDL levels also increase LDL levels. Increased cholesterol levels result in downregulation of native LDL receptors, thus inducing LDL uptake by receptor scavengers, ultimately leading to foam cell formation [37].

#### HDL

Table-3 shows that supplementation of MF and TF significantly affect (p<0.05) HDL levels of Cihateup ducks (R_1_, R_2_, and R_3_ groups). The treatment of TF only R5 shows the best results compared to controls and other treatments. This study is in accordance with that of Dono [38] who reports that the addition of turmeric at 0.35% level can increase HDL content and reduce total cholesterol, triglycerides, and LDL in broiler serum because curcumin is able to stimulate the activity of lipase-sensitive hormone. Curcumin increases lipid catabolism and reduces fat accumulation through its antioxidant. The combination of turmeric and thyme significantly (p<0.05) increases HDL until the 42^nd^ day of the study. This is because the compound of curcumin in turmeric can increase lipid catabolism and reduce fat accumulation by the presence of antioxidant properties that regulate and control the level of several hormones, inhibit the activity of several lipase enzymes, and increase the deposition of proteins in the body [6]. Adiputro *et al*. [33] reported that administration of the ethanolic extract of mangosteen pericarp increased HDL levels, starting at a dosage of 200 mg/kg BW. The main function of HDL is as a storage site of apolipoproteins C and E, which are needed in the catabolism of chylomicrons and VLDL, where apolipoprotein C is a cofactor of lipoprotein lipase, and apolipoprotein E is a ligand for LDL receptors.

### Abdominal fat

The data in Table-4 show that the supplementation with MF and TF significantly reduced (p<0.05) abdominal fat content (R_3_, R_4_, and R_5_ group) in Cihateup ducks. A previous study report that the addition of 0.5% TF significantly (p<0.01) reduces blood fat levels [16,39], abdominal fat, and liver percentages [40]. This is because curcumin has hypolipidemic and hypocholesterolemic properties that can decrease fat levels of broiler meat [41], stimulate bile secretion, and bile flow that can support liver health[38] and abdominal fat has a positive correlation with carcass weight and the percentage of abdominal fat in broilers reaches 2-3.13% at 4-8 weeks of slaughter period [11].

**Table-4:** Average abdominal fat of Cihateup ducks given ration contains mangosteen peel flour and turmeric flour of research results.

Treatment	Abdominal fat (g)	Percentage of abdominal fat (%)
R_0_(Positive control)	13.76±2.14^b^	1.12±0.21^b^
R_1_(2% MF)	14.11±3.48^b^	1.02±0.17^b^
R_2_(1.5% MF±0.5% TF)	12.87±3.07^b^	0.86±0.22^a^
R_3_(1% MF±1% TF)	9.44±1.35^a^	0.68±0.34^a^
R_4_(0.5% MF±1.5% TF)	7.33±0.89^a^	0.50±0.14^a^
R_5_(2% TF)	8.44±1.22^a^	0.62±0.33^a^
R_6_(Negative control)	14.75±3.54^b^	1.34±0.56^b^

Description - Superscript different within the same column shows a significant difference (p<0.05), MF=Mangosteen peel flour, TF=Turmeric flour

The treatment added with TF (Table-4) has the best effect compared to other treatments. Bile fluid is a greenish-yellow salt containing cholesterol, phospholipid, lecithin, and bile pigment. Bile contains a number of salts resulting from the mixture of sodium and potassium with bile acids (glycolic acid and taurocholate). These salts will mix with fat in the small intestine to form micelles. If the micelle is formed, it will reduce the tension between the fat surface and the mixing motion in the digestive tract gradually can break down the fat globules into finer particles so that fat can be digested [8,9]. Frandson [42] states that bile salts which are alkaline salts can help create an alkaline atmosphere in intestinal chyme. Bile salts that neutralize the acidity of the intestinal contents in the duodenal indentation area produce an alkaline state so that it can reach the appropriate pH, volume, and digestibility levels.

## Conclusion

The supplementation of MF and TF has a good effect on the production performance, lipid profile, and abdominal fat of Cihateup ducks. Xanthone and curcumin compounds on MF and TF combined at certain levels have the best impact on almost all test parameters compared to other treatments. The combination of both at a balanced level (1% each) is highly recommended to be applied as a supplement to poultry feed.

## Authors’ Contributions

The main research work was carried out and designed by AK and CHP. The research was carried out by AK and KRB. All authors drafted and revised the manuscript, read and approved the final manuscript.
